# Fabrication of Porous Poly(3-hydroxybutyrate-*co*-3-hydroxyhexanoate) Monoliths via Thermally Induced Phase Separation

**DOI:** 10.3390/polym8030066

**Published:** 2016-02-29

**Authors:** Takashi Tsujimoto, Nao Hosoda, Hiroshi Uyama

**Affiliations:** Department of Applied Chemistry, Graduate School of Engineering, Osaka University, Yamadaoka 2-1, Suita, Osaka 565-0871, Japan; n_hosoda@chem.eng.osaka-u.ac.jp (N.H.); uyama@chem.eng.osaka-u.ac.jp (H.U.)

**Keywords:** bio-based polymer, porous material, poly(3-hydroxyalkanoate), monolith, thermally induced phase separation, oil absorbent

## Abstract

This study deals with the fabrication of biodegradable porous materials from bacterial polyester, poly(3-hydroxybutyrate-*co*-3-hydroxyhexanoate) (P3HB3HH_x_), via thermally induced phase separation. P3HB3HH_x_ monoliths with topological porous structure were prepared by dissolution of P3HB3HH_x_ in dimethyl sulfoxide (DMSO) at 85 °C and subsequent quenching. The microstructure of the resulting P3HB3HH_x_ monoliths was changed by the P3HB3HH_x_ concentration of the polymer solution. Differential scanning calorimetry and polarized optical microscope analysis revealed that the P3HB3HH_x_ monoliths crystallized during phase separation and the subsequent aging. The mechanical properties, such as compression modulus and stress, of the monoliths depended on the 3-hydroxyhexanoate content of P3HB3HH_x_. Furthermore, the P3HB3HH_x_ monolith absorbed linseed oil in preference to water in a plant oil–water mixture. In combination with the biodegradable character of P3HB3HH_x_, the present study is expected to contribute to the development of bio-based materials.

## 1. Introduction

Polymeric materials with porous structure have attracted great attention due to their exciting properties, such as high specific surface area, high permeability, low density, and fast mass transfer performance [1,2,3]. Owing to these aforementioned properties, polymeric porous materials in the form of particles, fibers, films, membranes, and monoliths have been used in many fields such as packing, cushioning, thermal and/or mechanical insulation, electronics, catalytic supports, separation filters, and scaffolds for biomedical cell [4,5,6,7].

Up to now, several techniques have been developed to fabricate polymeric porous materials, including porogen leaching [8,9], expansion in high pressure gas [10,11], emulsion freeze-drying [12], 3D printing [13], freeze-drying [14,15], and phase separation [16,17,18,19,20]. The porogen leaching technique is a well-known method, and involves the casting of polymer/porogen composite, followed by washing out of the incorporated porogen. Various porogens such as poor solvent, salts, and polymers, are used to fabricate polymeric porous materials. The salt leaching method easily controls the pore sizes by changing the size of salts. The expansion in high pressure gas and emulsion freeze-drying techniques are other approaches for the fabrication of porous materials, but these methods often result in production of porous materials with closed cellular structure. The 3D printing method using ink-jet printing is useful for controlling the skeleton and pore structure of porous materials, whereas there are some problems such as the need for complicated and expensive apparatus.

Phase separation of polymer solution is one of the useful methods to fabricate porous materials with interconnected porous structure. There are several methods to induce phase separation such as thermally induced phase separation (TIPS) [21,22,23,24,25], solvent induced phase separation (SIPS) [26,27], and non-solvent induced phase separation (NIPS) [28,29,30,31]. Phase separation methods are based on thermodynamic demixing of a homogeneous polymer solution into a polymer-rich phase and polymer-lean phase. TIPS technique uses thermal energy to induce phase separation and makes it easy to control the morphology by adjusting various thermodynamic and kinetic parameters. In general, a homogeneous polymer solution is cooled to a temperature below spinodal solubility curve to produce a porous material. The TIPS method has often been used to fabricate porous materials for membranes and columns of separation.

Recently, the depletion of fossil fuel and the environmental issues of petroleum-based materials have derived a large interest in renewable bio-based products synthesized from natural resources [32]. The utilization of renewable resources as raw materials for monomers or polymers can contribute to sustainable development because it does not only decrease carbon dioxide emission but it also meets other advantages, such as high biodegradability and low toxicity.

A series of poly(3-hydroxyalkanoate)s (PHAs), including the poly(3-hydroxybutyrate) (P3HB) homopolymer and the related copolymers, are naturally occurring thermoplastics that behave as intercellular-carbon and energy-storage compound in microorganisms such as bacteria [33,34]. P3HB and the related copolymers can be decomposed in contact with divers hydrolytic enzymes, e.g., depolymerase, and microorganism, and their chemical structure and physical properties are similar to those of certain petroleum-based synthetic polymers. Therefore, they have potential applications in packaging, coating, and agricultural films [35,36]. Poly(3-hydroxybutyrate-*co*-3-hydroxyhexanoate) (P3HB3HH_x_) is one of the P3HB-related copolymers, and consists of randomly arranged 3-hydroxybutyrate (3HB) and 3-hydroxyhexanoate (3HH) units. Recently, P3HB3HH_x_ is industrially available in large volumes at a reasonable cost. P3HB3HH_x_ has a lower melting point and highly ductile properties compared with P3HB [37,38,39].

This study deals with the fabrication of novel bio-based porous materials from bacterial polyester. Bio-based porous materials are expected to be used for scaffolds and agricultural applications due to their biocompatibility and biodegradability. Some bio-based porous materials have been developed, but most of them were prepared from poly(lactic acid) or cellulose [40,41,42,43,44,45,46]. In this study, P3HB3HH_x_ monolith was prepared by TIPS method and the microstructure and properties of the resulting P3HB3HH_x_ monoliths were investigated. As far as we know, this study represents the first report on fabrication of porous PHA monoliths.

## 2. Experimental Section

### 2.1. Materials

P3HB3HH_x_s containing 6 and 11 mol % of 3HH (P3HB3HH_6_ and P3HB3HH_11_) were gifts from Kaneka Co. (Osaka, Japan). The weight-average molecular weights of P3HB3HH_6_ and P3HB3HH_11_, determined by size-exclusion chromatography (SEC), were 1.9 × 10^5^ and 1.7 × 10^5^, respectively. Polydispersity of P3HB3HH_6_ and P3HB3HH_11_ were 1.4 and 1.8, respectively. Dimethyl sulfoxide (DMSO) was purchased from Nacalai Tesque, Inc. (Kyoto, Japan). Other reagents and solvents were commercially available and were used as received.

### 2.2. Fabrication of P3HB3HH_x_ Monoliths

The fabrication of P3HB3HH_x_ monolith was accomplished as follows. P3HB3HH_6_ powder (200 mg) dissolved in 2 mL of DMSO at 85 °C for 15 min, and the solution was cooled at 20 °C. After 12 h, the solvent was replaced with water by immersing the resulting white solid into a large amount of water at room temperature. The sample was dried by lyophilization to give P3HB3HH_6_ monolith (100 g·L^−1^).

The P3HB3HH_6_ monoliths prepared in the solution with different P3HB3HH_6_ concentration and the P3HB3HH_11_ monoliths were prepared by similar procedure.

### 2.3. Measurements

Size-exclusion chromatographic (SEC) analysis was carried out at 40 °C by a SC8020 apparatus (Tosoh Co., Tokyo, Japan) with a refractive index detector and a TSKgel G4000_HHR_. The eluent used was chloroform at a flow rate of 1.0 mL·min^−1^. A calibration curve was obtained using polystyrene standards. Scanning electron microscopic (SEM) analysis was carried out using a SU3500 instrument (Hitachi High-Technologies Co., Tokyo, Japan) at an accelerating voltage of 15 kV. Nitrogen adsorption–desorption isotherm was conducted on a NOVA4200e (Quantachrome Co., Boynton Beach, FL, USA), and Brunauer–Emmett–Teller (BET) analysis was performed with the autosorb program. The thermal properties of the samples were measured under nitrogen atmosphere using a DSC6220 differential scanning calorimeter (DSC) (Hitachi High-Tech Science Co., Tokyo, Japan). The sample was heated from −50 to 175 °C at a heating rate of 10 °C·min^−1^, and the temperature was maintained for a duration of 2 min. Then, the sample was cooled to −50 °C at a cooling rate of 10 °C·min^−1^. The temperature was maintained for a duration of 2 min, and the sample was reheated to 180 °C at a heating rate of 10 °C·min^−1^. Polarized optical microscopic (POM) analysis was carried out using a BX51 microscope (Olympus Co., Tokyo, Japan) equipped with a MHS-2000 heating stage (Imoto Machinery Co. Ltd., Kyoto, Japan). The sample was placed on a glass slide at 85 °C, and subsequently cooled at room temperature. Compression test was performed by using a RheoStress 6000 (Thermo Fisher Scientific Inc., Waltham, MA, USA) with a cross-head speed of 1 mm·min^−1^.

## 3. Results and Discussion

### 3.1. Fabrication of P3HB3HH_x_ Monoliths

In this study, dimethyl sulfoxide (DMSO) was introduced as a solvent, and the P3HB3HH_x_ monoliths were fabricated using temperature-dependence solubility of P3HB3HH_x_ for DMSO. P3HB and the related copolymers are insoluble in DMSO at room temperature. However, they could be soluble in DMSO by heating. In the course of TIPS, polymer solution shows an upper critical solution temperature (UCST) behavior. The polymer solution exhibits phase separation below the UCST corresponding to cloud point. Phase separation is easily achieved by quenching the solution. The cloud points of the P3HB3HH_x_/DMSO solution increased with an increase in P3HB3HH_x_ concentration, and the polymer solution of P3HB3HH_6_ and P3HB3HH_11_ had very similar behaviors (Figure S1, Supplementary Materials). The cloud points of the P3HB3HH_6_ solution appeared about 10–15 °C higher than those of the P3HB3HH_11_ solution. This may be due to the differences in the molecular weight and the crystallinity of P3HB3HH_x_. In order to fabricate P3HB3HH_x_ monolith, P3HB3HH_x_ dissolved in DMSO at 85 °C, followed by cooling at 20 °C. The resulting white solid was washed with water and was dried by lyophilization to give the P3HB3HH_x_ monolith retaining the shape of the vessel.

**Figure 1:**
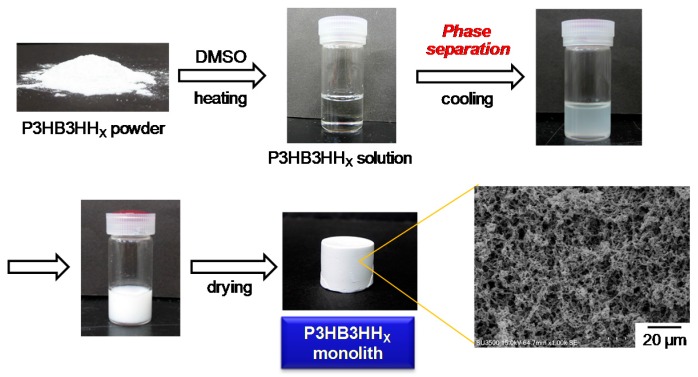
General procedure for fabrication of P3HB3HH_x_ monolith.

The general procedure for the fabrication of the P3HB3HH_x_ monoliths is illustrated in Figure 1. After quenching at 20 °C, the P3HB3HH_x_ polymer solution became gradually clouded, resulting in formation of a piece of white material. This behavior is presumed to be derived from the phase separation and the subsequent crystallization of P3HB3HH_x_. Solvent exchange with water after aging removed the residual DMSO from the skeleton derived from polymer-rich phase and is found to substantially reduce shrinkage during drying. In this process, the clear polymer solution was cooled below the UCST line and the Gibbs free energy of the system increased, which consequently resulted in phase separation. After the early-stage development of microstructure, the polymer-rich phase and polymer-lean phase continued to evolve to reduce the surface energy associated with interfacial area, and the crystallization of P3HB3HH_x_ proceeded in the polymer-rich phase. The resulting small crystals played a role as physical crosslinking points. In this study, we chose P3HB3HH_x_ among PHAs because of their highly ductile properties, and the mechanical properties and oil absorbency of the monolith was investigated.

### 3.2. Microstructure and Properties of P3HB3HH_x_ Monoliths

**Figure 2:**
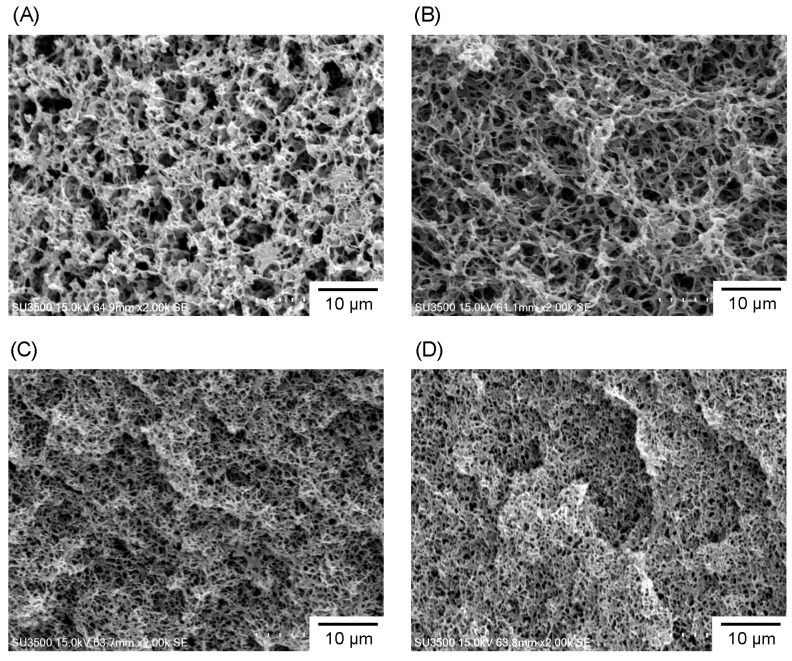
SEM images of P3HB3HH_6_ monoliths prepared from different polymer concentration in DMSO: (**A**) 50 g·L^−1^; (**B**) 100 g·L^−1^; (**C**) 150 g·L^−1^; and (**D**) 200 g·L^−1^.

In order to investigate the microstructure of the P3HB3HH_x_ monoliths, SEM analysis was carried out. Figure 2 displays the morphology of the P3HB3HH_6_ monoliths as a function of the polymer concentration. In all samples, the three-dimensional interconnected porous structures were observed. This characteristic structure was derived from a precursor gel and attributed to the phase separation of the polymer solution during the cooling process, in which polymer-rich regions contributed to the formation of skeletons. It is found that the pore size of the P3HB3HH_6_ monoliths becomes smaller with an increase in the polymer concentration. This is related to the higher cloud point of the polymer solution with higher concentration. Similar behaviors are observed in other polymer monolith prepared by TIPS method [16,17,20,25]. With the higher P3HB3HH_6_ concentration, some heterogeneous coagulations of P3HB3HH_6_ were observed. In SEM images of the P3HB3HH_11_ monoliths, homogeneous porous structures were also observed (Figure S2, Supplementary Materials). The pore and skeleton of the P3HB3HH_11_ monoliths were slightly thicker than those of the P3HB3HH_6_ monoliths.

**Figure 3:**
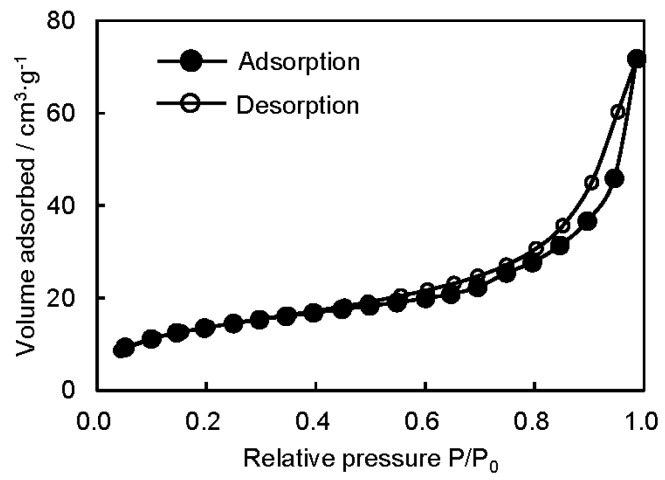
Typical nitrogen adsorption–desorption isotherm of P3HB3HH_6_ monolith (100 g·L^−1^).

**Figure 4:**
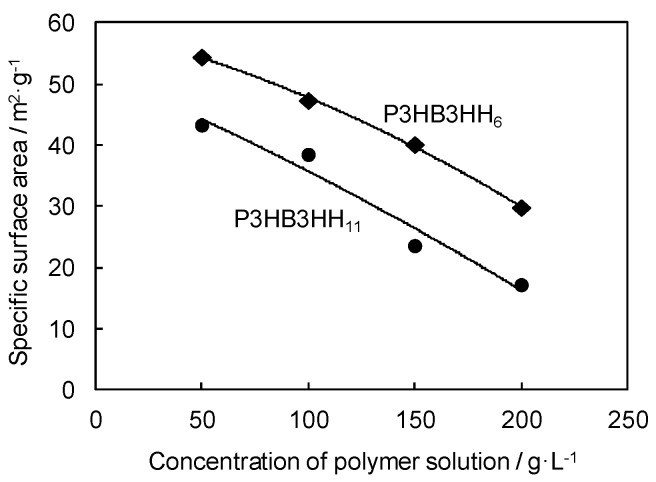
Relationship between concentration of polymer solution and specific surface area of P3HB3HH_x_ monoliths.

The typical nitrogen adsorption–desorption isotherm of the P3HB3HH_6_ monolith and the relationship between the polymer concentration and specific surface area of the P3HB3HH_x_ monoliths are shown in Figure 3 and Figure 4, respectively. The sharp nitrogen uptake near P_0_ and the hysteresis loop in the P/P_0_ range from 0.6 to 1.0 were observed, demonstrating the pore size distribution between mesopores and macropores (Figure 3). The specific surface area of the P3HB3HH_x_ monoliths, determined by BET analysis, decreased with an increase in the polymer concentration in DMSO solution, and varied from 17 to 55 m^2^·g^−1^ (Figure 4). The polymer concentration was one of the parameters for control of the microstructure of the P3HB3HH_x_ monoliths. The surface area of P3HB3HH_11_ monoliths was smaller than that of P3HB3HH_6_ monoliths. This result suggests that some mesopores of the monoliths are closed during the aging and drying processes due to low glass transition temperature (*T_g_*) of P3HB3HH_x_.

**Figure 5:**
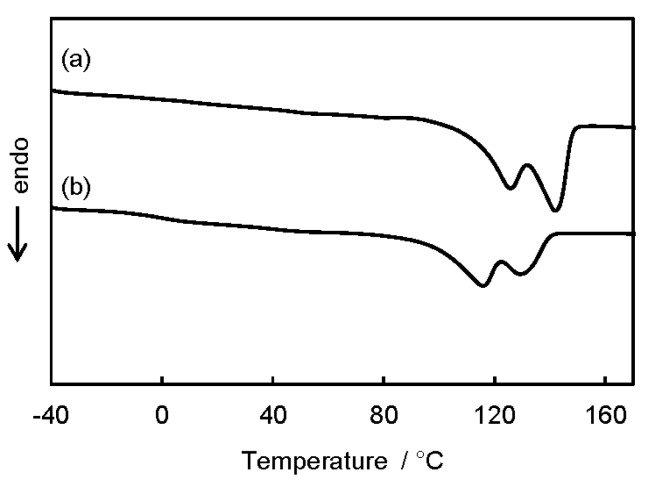
DSC curves of (**a**) P3HB3HH_6_ monolith (100 g·L^−1^); and (**b**) P3HB3HH_11_ monolith (100 g·L^−1^).

DSC measurement was performed to evaluate thermal properties of the P3HB3HH_x_ monoliths. Porous materials prepared by phase separation are shown to be semi-crystalline as a result of crystallization during phase separation. In the second heating curve for the P3HB3HH_6_ monolith, a glass transition was observed at approximately −3 °C, and the *T_g_* of the P3HB3HH_6_ monolith hardly changed compared with that of the P3HB3HH_6_ powder (*T_g_* = −2 °C), suggesting that the reaction and degradation of P3HB3HH_6_ did not proceed during the phase separation and the subsequent aging (Figure S3, Supplementary Materials). Moreover, the P3HB3HH_6_ monolith and the P3HB3HH_11_ monolith exhibited two broad melting peaks of P3HB3HH_x_ crystals in the first heating curves, demonstrating the melting-recrystallization-melting process (Figure 5). During the phase separation, DMSO diffused from polymer-rich phase to polymer-lean phase, and the crystallization of P3HB3HH_x_ proceeded.

**Figure 6:**
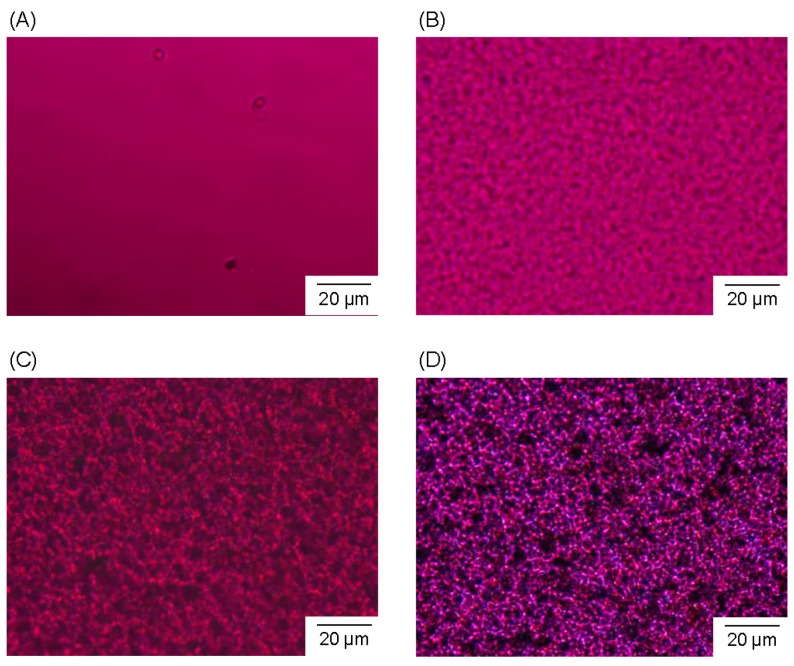
Polarized optical micrographs of fabrication process of the P3HB3HH_6_ monolith (100 g·L^−1^): (**A**) 0 min; (**B**) 5 min; (**C**) 60 min; and (**D**) one day after quenching.

Figure 6 shows the POM images of the fabrication process of the P3HB3HH_6_ monolith (100 g·L^−1^). The POM images were taken 0, 5, and 60 min as well as one day after the quenching from the P3HB3HH_6_/DMSO solution at 85 °C to room temperature. The P3HB3HH_6_/DMSO solution showed clear and homogeneous solution before the quenching (Figure 6A). It is found that the phase separation gradually proceeds and the porous structure is formed (Figure 6B,C). Moreover, the image taken one day after the quenching displayed that the skeleton consisted of the crystals of P3HB3HH_6_ (Figure 6D). This result is a characteristic proof that the crystallization of P3HB3HH_x_ proceeded during the phase separation and the subsequent aging. The nucleation and crystal growth were involved in the formation of the skeleton of the P3HB3HH_x_ monoliths in the polymer-rich phase, and the P3HB3HH_x_ crystals would play a crucial role in fixing of the porous structure of the monoliths.

**Figure 7:**
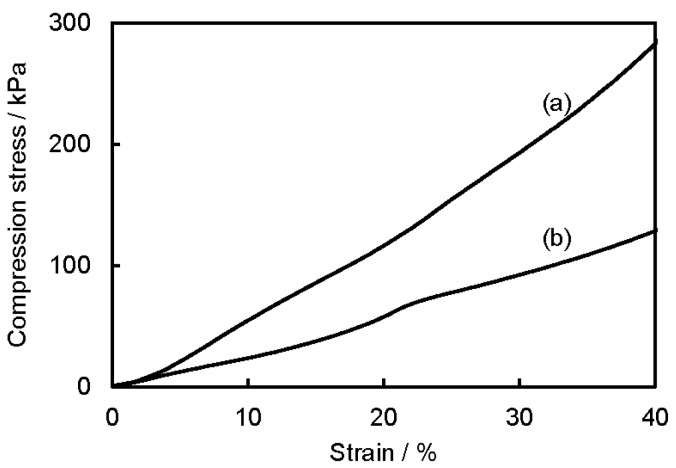
Compression stress–strain curves of (**a**) P3HB3HH_6_ monolith (100 g·L^−1^) and (**b**) P3HB3HH_11_ monolith (100 g·L^−1^).

The mechanical properties of P3HB3HH_x_ monoliths were measured with the selected sample, and Figure 7 shows the compression test results of the P3HB3HH_6_ monolith (100 g·L^−1^) and P3HB3HH_11_ monolith (100 g·L^−1^). For both monoliths, durable behaviors were observed, and the P3HB3HH_6_ monolith and P3HB3HH_11_ monolith were not fractured at even 40% deformation. The compression modulus and stress of 40% deformation of the P3HB3HH_6_ monolith were larger than those of the P3HB3HH_11_ monoliths, and the stress of 40% deformation of the P3HB3HH_6_ monolith was 285 kPa. This may be mainly governed by the crystallinity of P3HB3HH_x_ rather than their porosity or morphology.

**Figure 8:**
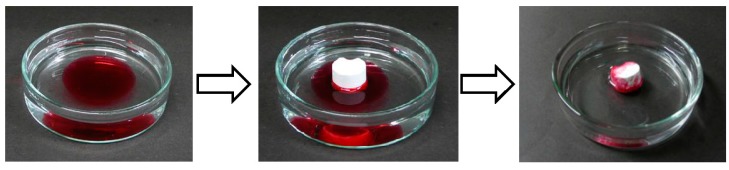
Separation of linseed oil layer on water surface using P3HB3HH_6_ monolith.

PHAs are known as hydrophobic polymers due to their aliphatic side chain. The oleophilicity in combination with hydrophobicity makes it a candidate for oil absorbent. Thus, an oil absorption test of the P3HB3HH_6_ monolith was demonstrated (Figure 8). When the P3HB3HH_6_ monolith (100 g·L^−1^) was brought into contact with a linseed oil layer containing Oil Red O drifting on water, the monolith absorbed the linseed oil colored with red dye from the water surface. This is due to low density and hydrophobicity of the P3HB3HH_6_ monolith. The P3HB3HH_6_ monolith was easily removed from the water surface, and clear water was left behind. This result indicates a high potential of the P3HB3HH_x_ monoliths for a candidate for biodegradable oil absorbent.

## 4. Conclusions

In this study, novel porous poly(3-hydroxyalkanoate) monoliths were successfully fabricated from their DMSO solution via thermally induced phase separation. P3HB3HH_x_ could dissolved in DMSO by heating, and subsequent quenching produced the P3HB3HH_x_ monolith with three-dimensional interconnected porous structure. The microstructure and the specific surface area of the P3HB3HH_x_ monoliths were easily controlled by the P3HB3HH_x_ concentration of the polymer solution. All of the P3HB3HH_x_ monoliths were found to be semi-crystalline, and the polarized optical microscopy showed the skeleton consisted of the crystals of P3HB3HH_x_. The mechanical properties, such as compression modulus and stress of the P3HB3HH_6_ monolith, were larger than those of P3HB3HH_11_. Furthermore, the P3HB3HH_x_ monolith exhibited good plant oil absorption capacity. Considering the high porosity, good mechanical properties, and oil absorbency of the P3HB3HH_x_ monoliths as well as the template-free and versatile fabrication procedure, this study provided a method for the design and fabrication of bio-based porous materials.

## Supplementary Materials

Supplementary materials can be found at www.mdpi.com/2073-4360/8/3/66/s1.
