# Supplementary material for: Fabrication of Porous Poly(3-hydroxybutyrate-co-3-hydroxyhexanoate) Monoliths via Thermally Induced Phase Separation
Source: Polymers (Basel). 2016 Feb 29;8(3):66. doi: 10.3390/polym8030066 (PMC6432561; doi:10.3390/polym8030066)
Supplement: Supplementary file 1 [file polymers-08-00066-s001.pdf]

Supplementary Materials: Fabrication of Porous Poly(3-hydroxybutyrate-*co*-3-hydroxyhexanoate) Monoliths via Thermally Induced Phase Separation

Takashi Tsujimoto *, Nao Hosoda and Hiroshi Uyama

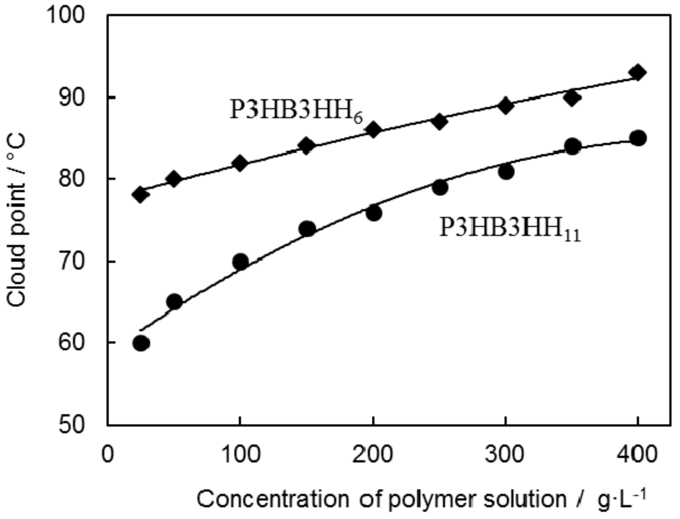

Figure S1. Cloud point curves of P3HB3HHx/DMSO solution.

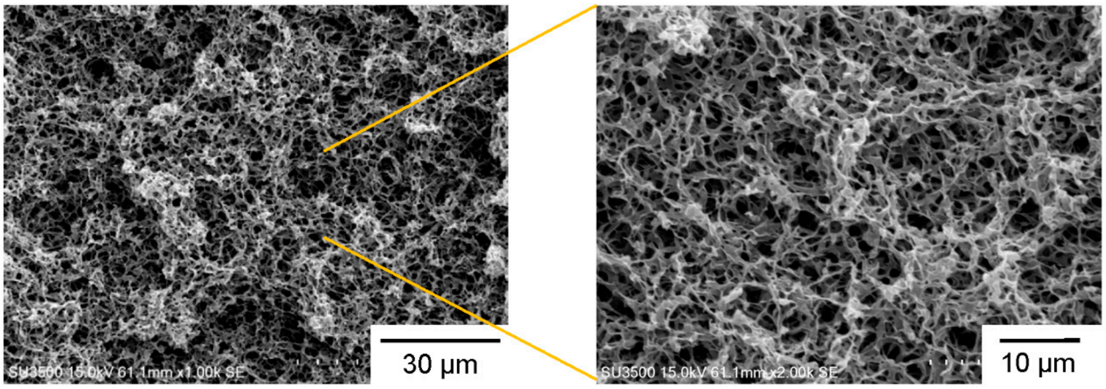

(**A**) P3HB3HH6 monolith

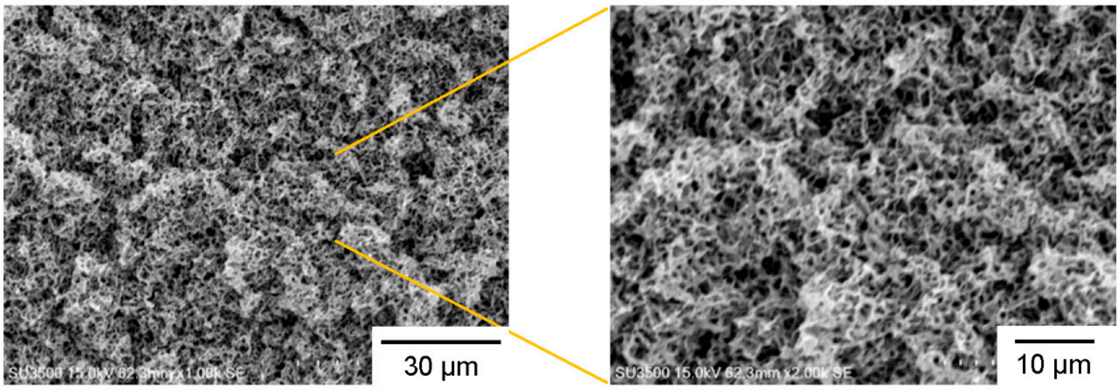

(**B**) P3HB3HH11 monolith

Figure S2. SEM images of (**A**) P3HB3HH6 monolith (100 g·L^{-1}) and (**B**) P3HB3HH11 monolith (100 g·L^{-1}).

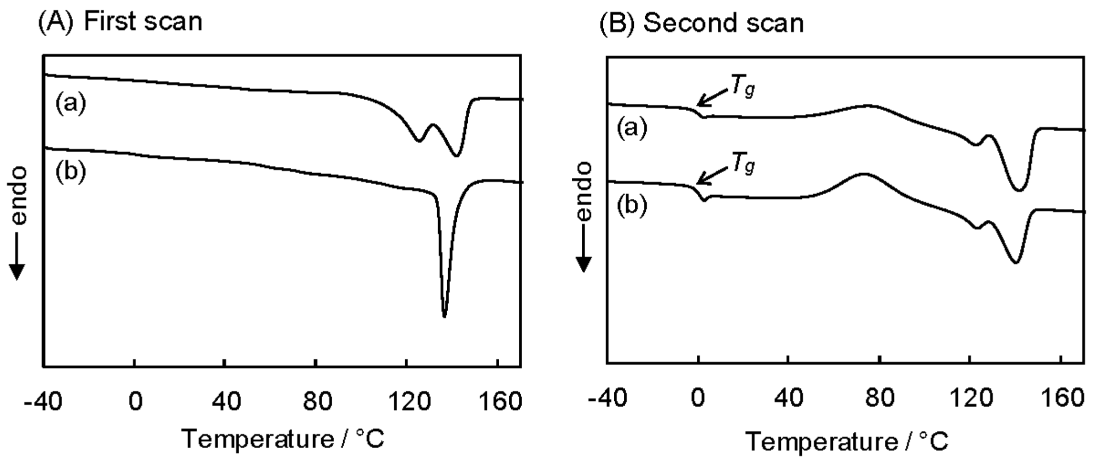

Figure S3. DSC curves of (a) P3HB3HH6 monolith (100 g·L^{-1}), and (b) P3HB3HH6 powder; (**A**) first heating scan, and (**B**) second heating scan.

© 2016 by the authors; licensee MDPI, Basel, Switzerland. This article is an open access article distributed under the terms and conditions of the Creative Commons by Attribution (CC-BY) license (http://creativecommons.org/licenses/by/4.0/).
